# The Association Between Stress Hyperglycemia Ratio and Short‐Term Outcomes After Successful Recanalization by Mechanical Thrombectomy in Acute Ischemic Stroke Patients

**DOI:** 10.1002/brb3.71062

**Published:** 2025-11-11

**Authors:** Xiaobo Li, Chao Jiang, Danni Chen, Wenyong Gao, Xianmin Wang

**Affiliations:** ^1^ Department of Neurology Northern Jiangsu People's Hospital Affiliated to Yangzhou University Yangzhou China; ^2^ Department of Neurosurgery Northern Jiangsu Peopleer Hospital Affiliated to Yangzhou University Yangzhou China; ^3^ Department of Neurophysiology Northern Jiangsu Peopleer Hospital Affiliated to Yangzhou University Yangzhou China

**Keywords:** acute ischemic stroke, mechanical thrombectomy, prognosis, stress hyperglycemia ratio, vascular recanalization

## Abstract

**Objective:**

Transient hyperglycemia, known as stress‐induced hyperglycemia, often occurs after acute ischemic events. This study aims to examine the relationship between stress hyperglycemia (SHG) and short‐term outcomes following successful recanalization by mechanical thrombectomy (MT) in patients with acute ischemic stroke (AIS), providing evidence for predicting short‐term prognosis.

**Methods:**

This study included AIS patients who underwent MT and achieved successful recanalization between July 2018 and October 2023 at Northern Jiangsu People's Hospital. Stress hyperglycemia ratio (SHR) was calculated using fasting blood glucose (FBG) and glycated hemoglobin (HbA1c) levels at admission to measure SHG. The primary outcome was a poor prognosis at 90 days. Secondary outcomes included early neurological deterioration (END), hemorrhagic transformation (HT), symptomatic intracranial hemorrhage (sICH), and 90‐day death. Multivariable logistic regression analysis was performed to evaluate the association between SHR and short‐term prognosis.

**Results:**

A total of 394 patients were enrolled. The poor prognosis group had significantly higher SHR than the favorable prognosis group [1.00 (0.87, 1.22) versus 0.82 (0.70, 0.99); *p* < 0.001]. Multivariable logistic regression analysis confirmed that SHR was independently associated with poor prognosis at 90 days (*p* < 0.05).

**Conclusion:**

SHR is associated with both the severity of the condition at admission and a 90‐day poor prognosis in AIS patients after successful recanalization by mechanical thrombectomy.

## Graphical abstract

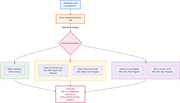

## 1 Introduction

Acute ischemic stroke (AIS) is the most common type of stroke and a leading cause of severe disability and death worldwide. Among adults aged 40 years old and older, the estimated overall prevalence, incidence, and mortality rate of stroke in 2020 were 2.6%, 505.2 per 100,000 person‐years, and 343.4 per 100,000 person‐years, respectively, indicating 17.8 million cases of stroke, 3.4 million new strokes, and 2.3 million stroke‐related deaths in China (Tu et al. 2023). The risk of stroke increases significantly in patients with hyperglycemia, particularly in AIS cases. Approximately 40% of AIS patients present with hyperglycemia at admission, and this phenomenon occurs not only in diabetic patients but also in non‐diabetic patients who may experience elevated blood glucose levels following AIS (Johnston et al. 2019). The transient increase in blood glucose observed in critically ill patients, including those with stroke, is termed stress hyperglycemia (SHG), which typically resolves after recovery from the acute injury (Harp et al. 2016). SHG is a frequent pathophysiological response in critically ill patients and is strongly associated with clinical outcomes. Snarska (2017) reported that SHG at admission is linked to worse clinical outcomes and increased in‐hospital mortality in both ischemic and hemorrhagic stroke patients. Given the distinct pathophysiological mechanisms of chronic hyperglycemia and SHG, a reliable measure for assessing SHG has been developed: the stress hyperglycemia ratio (SHR). SHR is calculated as the blood glucose concentration at admission divided by the estimated average glucose concentration based on HbA1c levels (Roberts et al. 2015). This calculation adjusts for baseline glucose levels, offering a more accurate assessment of SHG and the condition of critically ill patients.

Large vessel occlusion (LVO) occurs in approximately 24%–38% of AIS cases. Compared to non‐LVO patients, AIS caused by LVO leads to larger infarct areas, more severe neurological deficits, and worse long‐term outcomes. Previous studies have shown that the risk of death is more than twice as high in LVO‐related AIS compared to non‐LVO AIS (Malhotra et al. 2017; Heldner et al. 2013; Kodumuri et al. 2016; Daneshvari and Johansen 2021). With advancements in medicine and technology, mechanical thrombectomy has become a primary treatment option in the hyperacute phase for LVO stroke patients. However, LVO‐related AIS is more severe than other types of AIS. Despite the effectiveness of endovascular therapy in achieving successful recanalization, many patients continue to experience poor outcomes (Deng et al. 2022). Thus, it is essential to identify predictive markers for neurological deterioration and adverse outcomes in LVO patients following successful MT. Currently, limited research has explored the relationship between SHR and the outcomes of AIS patients after successful recanalization. We aimed to examine the association between SHR and adverse outcomes, as well as short‐term prognosis, to evaluate the predictive value of SHR for short‐term outcomes.

## 2 Materials and Methods

### 2.1 Study Population

This study included 394 AIS patients treated at Northern Jiangsu People's Hospital from July 2018 to October 2023. All patients underwent successful MT, achieving a modified Thrombolysis in the Cerebral Infarction (mTICI) grade of 2b or 3. The study was approved by the Medical Ethics Committee of Northern Jiangsu People's Hospital (**Ethics Approval Number: 2024ky312**). Due to its retrospective study, informed consent was waived. The study protocol adhered to the ethical guidelines of the Declaration of Helsinki.

Inclusion criteria: (1) age ≥18 years, (2) confirmed diagnosis of AIS with digital subtraction angiography (DSA) evidence of large vessel occlusion, (3) pre‐stroke modified Rankin Scale (mRS) score of 0, (4) Admission Alberta Stroke Program Early Computed Tomography Score (ASPECTS) ≥ 6, (5) onset‐to‐puncture time ≤ 24 h, and (6) underwent MT with successful recanalization (mTICI grade of 3 or 2b).

Exclusion criteria: (1) severe hepatic or renal dysfunction; (2) stroke due to autoimmune diseases or cerebral venous sinus thrombosis; (3) recent severe trauma, infection, or malignancy; (4) corticosteroid use, severe anemia, hyperthyroidism, or hypothyroidism; and (5) missing baseline data or loss to follow‐up.

### 2.2 Data Collection

Baseline demographic and clinical data were collected, including age, gender, and medical history (hypertension, diabetes, coronary artery disease, atrial fibrillation, previous stroke, smoking, and drinking). Laboratory tests conducted within 24 h of admission included FBG, HbA1c, and other relevant assessments. Imaging data [cranial computed tomography (CT) and CT angiography (CTA)] were also obtained. Stroke severity was evaluated by trained neurologists using the NIHSS scores at admission. MT‐related data included time of symptom onset, hospital arrival, femoral artery puncture, recanalization time, number of attempts, collateral circulation, and affected vessel (anterior or posterior circulation). Assessment of SHG: SHR was calculated using the formula: SHR = [FBG / (1.59 × HbA1c − 2.59)] (Roberts et al. 2015). Fasting venous blood samples were collected within 24 h of admission for FBG and HbA1c measurement. All tests were performed in the laboratory of Northern Jiangsu People's Hospital.

### 2.3 Outcome Measures

All patients underwent cranial CT and MRI imaging before and after the procedure. Outcomes were assessed via telephone follow‐up. Primary outcome: Poor prognosis at 90 days, defined as an mRS score of 3–6. Secondary outcomes: END: Increase in NIHSS score of ≥ 4 points within 72 h post‐procedure compared to admission. HT: No hemorrhagic foci on preoperative CT, with parenchymal hemorrhage observed on follow‐up CT at 24 h or 7 days. sICH: Parenchymal hemorrhage on follow‐up CT within 7 days, accompanied by an increase of ≥ 4 points in the NIHSS score compared to preoperative assessment. Death: Includes in‐hospital deaths following resuscitation and deaths recorded during the 90‐day follow‐up.

### 2.4 Statistical Analysis

Data analysis was performed using SPSS software (version number 26.0; IBM company). Continuous variables with normal distribution are expressed as mean ± standard deviation and compared using independent t‐tests or ANOVA. Non‐normally distributed variables are presented as median (interquartile range) and compared using non‐parametric tests. Categorical data are shown as counts (percentages) and analyzed using the chi‐square test or Fisher's exact test. Variables with *p* < 0.05 in univariate analysis were included in multivariable logistic regression to identify independent risk factors affecting poor prognosis in AIS patients after successful recanalization by MT. The association between SHR and patient outcomes was further assessed. Receiver operating characteristic (ROC) curves were plotted to evaluate the predictive value of SHR for a 90‐day poor prognosis, with the area under the curve (AUC) calculated. A two‐sided *p* < 0.05 was considered statistically significant.

## 3 Results

### 3.1 Participant Characteristics

**FIGURE 1:**
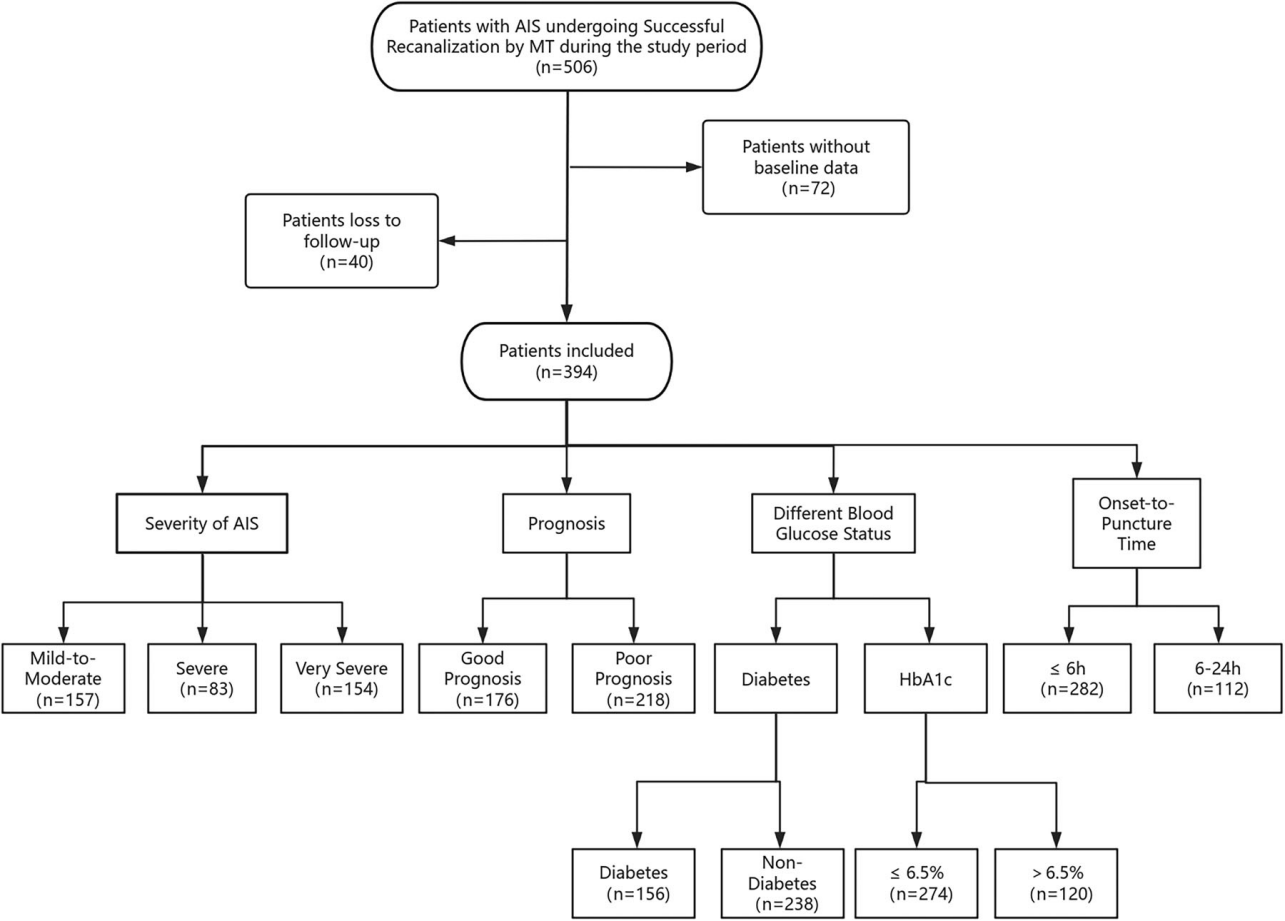
Flow diagram of the study. AIS, acute ischemic stroke; MT, mechanical thrombectomy; HbA1c, glycated hemoglobin.

This study retrospectively analyzed data from 394 AIS patients who underwent MT and achieved successful recanalization at Northern Jiangsu People's Hospital between July 2018 and October 2023 (see Figure 1). Among these patients, 227 (57.6%) were male. The cohort included 270 patients (68.5%) with hypertension, 161 (40.9%) with atrial fibrillation, 58 (14.7%) with coronary artery disease, 54 (13.7%) with previous stroke, 118 (29.9%) with a history of smoking, and 77 (19.5%) with a history of drinking. Based on admission NIHSS scores, patients were divided into three categories: the mild‐to‐moderate stroke group (NIHSS scores ≤ 15) with 157 patients (39.8%); the severe stroke group (NIHSS scores: 16–20) with 83 patients (21.1%); and the very severe stroke group (NIHSS scores ≥ 21) with 154 patients (39.1%). A total of 156 patients (39.6%) had a history of diabetes, while 238 (60.4%) did not. The cohort was further stratified by HbA1c levels: 120 patients (30.5%) had HbA1c > 6.5%, and 274 patients (69.5%) had HbA1c patients (39.1%). A total of 156 patients (39.6%) had a history of diabetes, while 238 (60.4%) did not. The cohort was further stratified by HbA1c levels:prognosis. Refer to Table 1 for details.

**TABLE 1:** General characteristics of the patients.

Variable	
Age (years), M (IQR)	69 (60.75, 75)
Male, n (%)	227 (57.6)
Hypertension, n (%)	270 (68.5)
Diabetes, n (%)	156 (39.6)
Atrial fibrillation, n (%)	161 (40.9)
Coronary artery disease, n (%)	58 (14.7)
Previous stroke, n (%)	54 (13.7)
Smoking, n (%)	118 (29.9)
Drinking, n (%)	77 (19.5)
NIHSS score at admission, M (IQR)	18 (12, 26)
Baseline SBP (mmHg), M (IQR)	149.5 (136, 164)
Baseline DBP (mmHg), M (IQR)	84 (76, 93)
ASPECTS, M (IQR)	9 (8,9)
Intravenous thrombolysis, n (%)	96 (24.4)
OTP time (hours), M (IQR)	4.88 (3.65, 6.27)
OTR time (hours), M (IQR)	6.47 (5.17, 8.01)
ATP time(hours), M (IQR)	2.38 (1.85, 3.22)
ATR time(hours), M (IQR)	4.03 (3.33, 5.00)
Number of passes, mean (SD)	1.93 ± 0.97
ASITN/SIR grade, n (%)	
3–4	70 (17.8)
0–2	324 (82.2)
TOAST, n (%)	
LAA,	233 (59.1)
CE	161 (40.9)
Anterior circulation occlusion, n (%)	293 (74.4)
Laboratory tests	
FBG(mmol/L), M (IQR)	6.54 (5.41, 8.92)
HbA1c (%), M (IQR)	5.9 (5.5, 6.9)
HbA1c > 6.5%	120 (30.5%)
HbA1c ≤ 6.5%	274 (69.5%)
RBC (10 ^ 12/L), mean (SD)	4.52 ± 0.61
NC (10 ^ 9/L), M (IQR)	5.38 (4.01, 7.35)
LC (10 ^ 9/L), M (IQR)	1.35 (0.96, 1.87)
PLC (10 ^ 9/L), M (IQR)	176 (142.75, 218.25)
D‐dimer (mg/L), M (IQR)	0.69 (0.36, 1.48)
TG (mmol/L), M (IQR)	1.18 (0.87, 1.56)
TC (mmol/L), M (IQR)	4.15 (3.65, 4.82)
LDL‐C (mmol/L), M (IQR)	2.37 (1.78, 2.92)
HDL‐C (mmol/L), M (IQR)	1.12 (0.97, 1.36)
APTT (s), M (IQR)	28.3 (26.38, 30.83)
FIB (g/L), M (IQR)	2.69 (2.27, 3.19)
PT (s), M (IQR)	11.75 (11.2, 12.4)
SHR, M (IQR)	0.93 (0.77, 1.13)
Severity of AIS, n (%)	
Mild‐to‐moderate (NIHSS score ≤ 15)	157 (39.8)
Severe (NIHSS score: 16–20)	83 (21.1)
Very severe (NIHSS score ≥ 21)	154 (39.1)
END, n (%)	71 (18)
HT, n (%)	171 (43.4)
sICH, n (%)	56 (14.2)
90‐day death, n (%)	50 (12.7)
90‐day poor prognosis, n (%)	218 (55.3)

**Abbreviations**: APTT, activated partial thromboplastin time; ATP, arrival‐to‐puncture; ATR, arrival‐to‐recanalization; CE, cardioembolism; DBP, diastolic blood pressure; FIB, fibrinogen; HDL‐C, high‐density lipoprotein cholesterol; LAA, large‐artery atherosclerosis; LC, lymphocyte count; LDL‐C, low‐density lipoprotein cholesterol; NC, neutrophil count; OTP, onset‐to‐puncture; OTR, onset‐to‐recanalization; PLC, platelet count; PT, prothrombin time; RBC, red blood cell count; SBP, systolic blood pressure; TC, total cholesterol; TG, triglyceride; TOAST, trial of org 10 172 in acute stroke treatment.

### 3.2 Correlation Analysis Between SHR and Severity of AIS in Patients

Significant differences were observed among the three severity groups in terms of age, diabetes, ASPECTS, smoking, anterior circulation occlusion, FBG, and LDL‐C (*p* < 0.05). The very severe stroke group exhibited higher SHR compared to the other groups [0.98 (0.78, 1.24) vs. 0.92 (0.75, 1.09) vs. 0.91 (0.77, 1.04); *p* = 0.044] (see Table 2). Multivariable ordinal logistic regression analysis indicated that SHR was independently associated with stroke severity (*p* = 0.022). Refer to Table 3 for details.

**TABLE 2:** Comparison of baseline characteristics of AIS patients across different stroke severity levels after successful recanalization by MT.

Variable	Mild‐to‐moderate(n = 157)	Severe (n = 83)	Very severe(n = 154)	*p‐*value
				
Male, n (%)	88 (56.1)	48 (57.8)	91 (59.1)	0.862
Hypertension, n (%)	100 (63.7)	57 (68.7)	113 (73.4)	0.184
Diabetes, n (%)	48 (30.6)	34 (41)	74 (48.1)	0.007
Atrial fibrillation, n (%)	63 (40.1)	37 (44.6)	61 (39.6)	0.737
Coronary artery disease, n (%)	20 (12.7)	10 (12)	28 (18.2)	0.296
Previous stroke, n (%)	19 (12.1)	8 (9.6)	27 (17.5)	0.182
Smoking, n (%)	54 (34.4)	30 (36.1)	34 (22.1)	0.023
Drinking, n (%)	38 (24.2)	17 (20.5)	22 (14.3)	0.085
Baseline SBP (mmHg), M (IQR)	149 (135, 162)	146 (134.5, 160)	150 (137, 166)	0.267
Baseline DBP (mmHg), M (IQR)	85 (75, 92)	86 (78.5, 93)	81.5 (75, 93)	0.474
Aspects, M (IQR)	9 (8, 10)	9 (8, 9)	8 (7, 9)	<0.001
TOAST, n (%)				0.204
LAA,	96 (61.1)	42 (50.6)	95 (61.7)	
CE	61 (38.9)	41 (49.4)	59 (38.3)	
Anterior circulation occlusion	129 (82.2)	69 (83.1)	95 (61.7)	<0.001
Laboratory tests				
FBG (mmol/L), M (IQR)	6.38 (5.37, 8.03)	6.29 (5.42, 8.38)	7.1 (5.7, 10.13)	0.007
HbA1c (%), M (IQR)	5.9 (5.6, 6.5)	6 (5.5, 7)	6.05 (5.5, 7.3)	0.620
RBC (10 ^ 12/L), mean (SD)	4.56 ± 0.61	4.44 ± 0.61	4.53 ± 0.62	0.377
NC (10 ^ 9/L), M (IQR)	5.35 (3.94, 6.81)	5.61 (3.9, 7.50)	5.29 (4.09, 7.53)	0.505
LC (10 ^ 9/L), M (IQR)	1.45 (1, 2.03)	1.25 (1.01, 1.63)	1.26 (0.9, 1.96)	0.206
PLC (10 ^ 9/L), M (IQR)	178 (146, 218)	170 (128, 223.5)	177 (142, 217)	0.553
D‐dimer (mg/L), M (IQR)	0.62 (0.33, 1.28)	0.84 (0.37, 1.77)	0.67 (0.36, 1.48)	0.305
TG (mmol/L), M (IQR)	1.15 (0.87, 1.62)	1.16 (0.89, 1.45)	1.21 (0.89, 1.56)	0.970
TC (mmol/L), M (IQR)	4.27 (3.78, 4.93)	4.08 (3.59, 4.74)	4.07 (3.55, 4.71)	0.147
LDL‐C (mmol/L), M (IQR)	2.55 (1.94, 3.12)	2.29 (1.77, 2.77)	2.26 (1.67, 2.85)	0.011
HDL‐C (mmol/L), M (IQR)	1.14 (0.96, 1.39)	1.12 (1.01, 1.42)	1.11 (0.94, 1.33)	0.318
APTT (s), M (IQR)	28.8 (26.4, 31)	28.2 (26.8, 31)	28 (26.2, 30.7)	0.597
FIB (g/L), M (IQR)	2.8 (2.32, 3.21)	2.64 (2.26, 3.14)	2.68 (2.25, 3.2)	0.541
PT (s), M (IQR)	11.7 (11.1, 12.3)	11.7 (11.3, 12.4)	11.8 (11.3, 12.6)	0.253
SHR, M (IQR)	0.91 (0.77, 1.04)	0.92 (0.75, 1.09)	0.98 (0.78, 1.24)	0.044

**TABLE 3:** Multivariable ordinal logistic regression analysis of AIS patients across different stroke severity levels after successful recanalization by MT.

Variable	OR	95%CI	*p*‐value
Age	1.025	1.007–1.044	0.007
Diabetes	1.820	1.204–2.748	0.004
Smoking	0.661	0.424–1.031	0.068
ASPECTS	0.608	0.506–0.730	< 0.001
LDL‐C	0.756	0.594–0.962	0.023
Anterior circulation occlusion	0.328	0.205–0.523	< 0.001
SHR*	1.069	1.010–1.133	0.022

SHR* = SHR × 10.

### 3.3 Correlation Analysis Between SHR and 90‐Day Prognosis in AIS Patients after Successful Recanalization by MT

**FIGURE 2:**
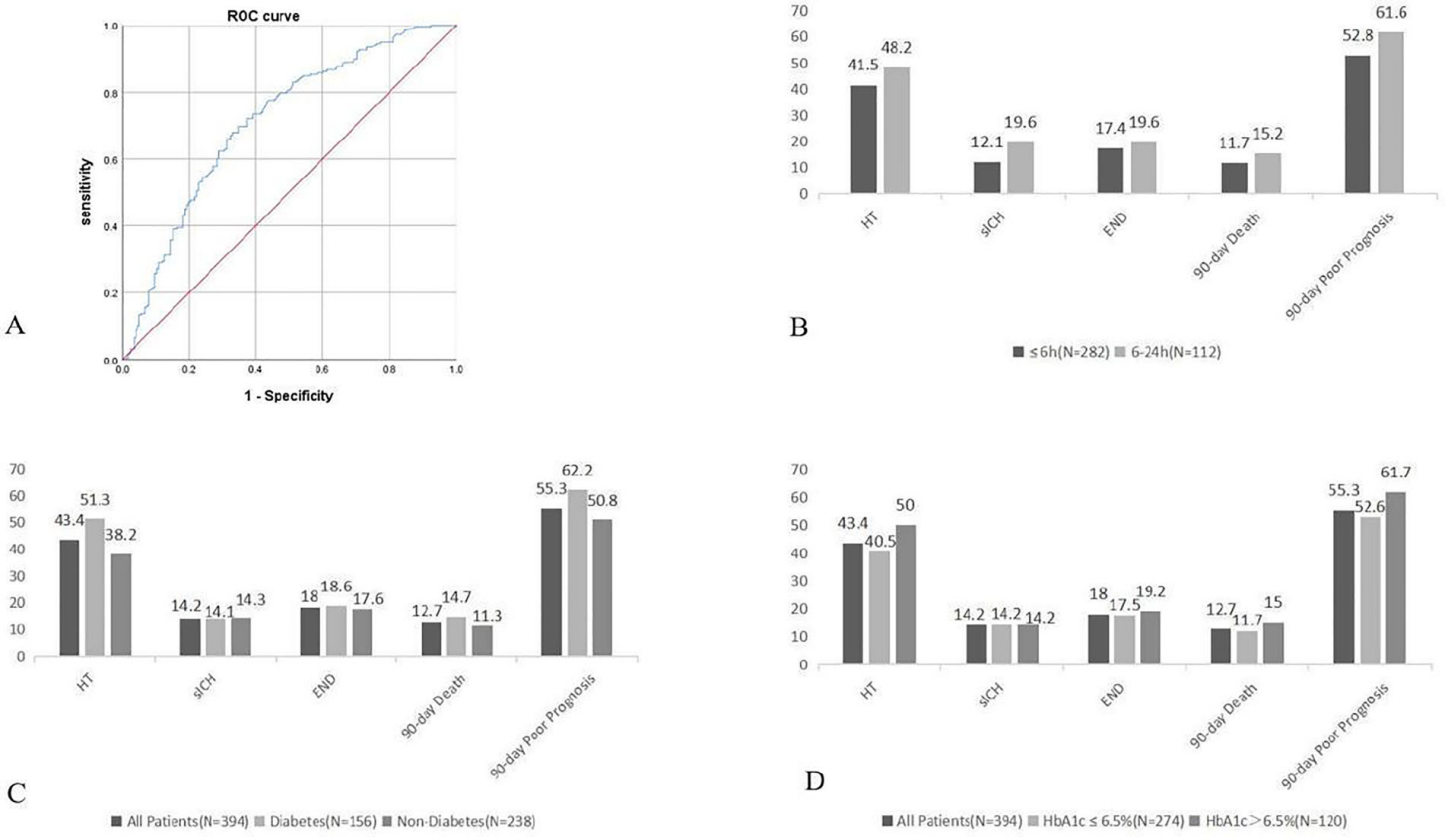
(A) ROC curve for predicting 90‐day poor prognosis using SHR in AIS patients after successful recanalization by MT. (B)–(D) Rates of 3‐month poor outcome: (B) in the OTP time ≤ 6 h group and the OTP time between 6 and 24 h group and (C) in the diabetes group and the non‐diabetes group D.in the HbA1c > 6.5% group and HbA1c ≤ 6.5% group.

Within the study cohort, the SHR was significantly higher in the poor prognosis group compared to the good prognosis group [10.0 (8.7, 12.2) vs 8.2 (7.0, 9.9) 1.00 (0.87, 1.22) vs 0.82 (0.70, 0.99); *p* < 0.001] (see Table 4). Multivariable logistic regression analysis revealed that SHR was an independent predictor of 90‐day poor prognosis (*p* < 0.001). Refer to Table 5 for details. The predictive value of SHR for 90‐day poor prognosis in AIS patients following successful MT was assessed using an ROC curve. The analysis indicated an AUC of 0.710 (*p* < 0.05). Based on the Youden Index, the optimal cutoff value for SHR in predicting 90‐day poor prognosis was 0.91, yielding a sensitivity of 69.7% and a specificity of 65.3% (see Figure 2A). These results suggest that SHR has significant predictive value for identifying 90‐day poor outcomes in AIS patients after successful recanalization.

**TABLE 4:** Comparison of baseline characteristics between good and poor prognosis groups in AIS patients after successful recanalization by MT.

Variable	Good prognosis (n = 176)	Poor prognosis (n = 218)	*p‐*value
Age (years), M (IQR)	66 (57.5, 73)	70 (64, 76)	< 0.001
Male, n (%)	107 (60.8)	120 (55)	0.251
Hypertension, n (%)	107 (60.8)	163 (74.8)	0.003
Diabetes, n (%)	59 (33.5)	97 (44.5)	0.027
Atrial fibrillation, n (%)	66 (37.5)	95 (43.6)	0.222
Coronary artery disease, n (%)	24 (13.6)	34 (15.6)	0.585
Previous stroke, n (%)	22 (12.5)	32 (14.7)	0.532
Smoking, n (%)	63 (35.8)	55 (25.2)	0.023
Drinking, n (%)	41 (23.3)	35 (16.5)	0.091
NIHSS score at admission, M (IQR)	15 (10, 22)	20 (14, 29)	< 0.001
Baseline SBP (mmHg), M (IQR)	148 (136, 160)	150 (136, 169)	0.066
Baseline DBP (mmHg), M (IQR)	83.5 (77, 92)	85 (75, 95)	0.984
ASPECTS, M (IQR)	9 (8, 10)	8 (7, 9)	< 0.001
Intravenous thrombolysis, n (%)	47 (26.7)	49 (22.5)	0.331
OTP time (hours), M (IQR)	4.82 (3.55, 6.0)	4.91 (3.67, 6.50)	0.343
OTR time (hours), M (IQR)	6.19 (4.85, 7.66)	6.78 (5.42, 8.31)	0.010
ATP time (hours), M (IQR)	2.30 (1.77, 3.22)	2.50 (1.88, 3.18)	0.259
ATR time (hours), M (IQR)	3.88 (3.01, 4.65)	4.28 (3.43, 5.18)	0.001
Number of passes, mean (SD)	1.77 ± 0.87	2.06 ± 1.02	0.002
ASITN/SIR grade, n (%)			< 0.001
3–4	52 (29.5)	18 (8.3)	
0–2	124 (70.5)	200 (91.7)	
TOAST, n (%)			0.692
LAA,	106 (60.2)	127 (58.3)	
CE	70 (39.8)	91 (41.7)	
Anterior circulation occlusion n (%)	131 (74.4)	162 (74.3)	0.978
Laboratory tests			
FBG (mmol/L), M (IQR)	5.77 (5.06, 7.09)	7.24 (6.10, 10.23)	< 0.001
HbA1c (%), M (IQR)	5.90 (5.50, 6.70)	6.05 (5.50, 7.00)	0.171
RBC (10 ^ 12/L), mean (SD)	4.57 ± 0.60	4.49 ± 0.63	0.174
NC (10 ^ 9/L), M (IQR)	5.35 (3.99, 7.17)	5.40 (4.06, 7.52)	0.637
LC (10 ^ 9/L), M (IQR)	1.36 (0.94, 1.95)	1.35 (0.98, 1.84)	0.915
PLC (10 ^ 9/L), M (IQR)	177 (143.5, 222)	175 (140, 218)	0.562
D‐dimer (mg/L), M (IQR)	0.51 (0.32, 1.17)	0.86 (0.38, 1.84)	0.001
TG (mmol/L), M (IQR)	1.21 (0.86, 1.49)	1.15 (0.90, 1.56)	0.856
TC (mmol/L), M (IQR)	4.19 (3.59, 4.75)	4.13 (3.70, 4.90)	0.564
LDL‐C (mmol/L), M (IQR)	2.32 (1.77, 2.84)	2.46 (1.78, 2.93)	0.159
HDL‐C (mmol/L), M (IQR)	1.10 (0.96, 1.38)	1.14 (0.97, 1.36)	0.724
APTT (s), M (IQR)	28.85 (26.15, 31)	28.1 (26.4, 30.7)	0.27
FIB (g/L), M (IQR)	2.70 (2.28, 3.23)	2.67 (2.27, 3.18)	0.389
PT (s), M (IQR)	11.85 (11.25, 12.35)	11.7 (11.2, 12.40)	0.451
SHR, M (IQR)	0.82 (0.70, 0.99)	1.00 (0.87, 1.22)	< 0.001

**TABLE 5:** Multivariable logistic regression analysis of poor prognosis in AIS patients after successful recanalization by MT.

Variable	OR	95%CI	*p*‐value
Age	1.036	1.013–1.06	0.002
Hypertension	1.615	0.967–2.698	0.067
Diabetes	1.539	0.935–2.532	0.09
Smoking	0.859	0.507–1.454	0.571
ASPECTS	0.765	0.608–0.963	0.022
NIHSS score at admission	1.029	1.001–1.058	0.045
ATR time	1.235	1.033–1.477	0.02
OTR time	1.033	0.964–1.107	0.359
Number of passes	1.354	1.055–1.737	0.017
ASITN/SIR grade	0.22	0.111–0.437	< 0.001
D‐dimer	1.702	0.965–1.192	0.196
SHR*	1.22	1.115–1.334	< 0.001

SHR* = SHR × 10.

### 3.4 Correlation Analysis of Adverse Outcomes and Prognosis in Different Time Windows for MT in AIS Patients

Among the included patients, rates of 3‐month poor outcomes (HT, sICH, END, 90‐day death, and prognosis) in the onset to puncture time ≤ 6 h group and the onset to puncture time between 6 and 24 h group are reported in Figure 2B.

Univariate analysis was performed, and indicators with *p* < 0.05 were selected for multivariable logistic regression analysis. The results indicated that in the OTP time ≤ 6 h group, SHR was an independent risk factor for sICH, END, 90‐day death, and poor prognosis in AIS patients after successful MT (*p* < 0.05). However, SHR was not significantly associated with HT (*p* > 0.05). In the OTP time between 6 and 24 h group, SHR was an independent risk factor for sICH, 90‐day death, and poor prognosis (*p* < 0.05), but showed no significant association with HT or END (*p* > 0.05). Refer to Table 6 for detailed results.

**TABLE 6:** Multivariable Regression Analysis of SHR and Adverse Outcomes in AIS Patients Across Different Time Windows.

Outcome	Onset‐to‐puncture time		OR	95%CI	*p‐*value
HT					
	≤Tlue	1.059	0.977–1.148	0.166	
	6–24h		1.097	0.939–1.28	0.243
sICH					
	≤ICH3	1.142	1.041–1.252	0.005	
	6–24h		1.231	1.036–1.463	0.018
END					
	≤ND18	1.173	1.076–1.279	< 0.001	
	6–24h		1.149	0.986–1.339	0.075
90‐day death					
	≤athh	1.107	1.009–1.215	0.032	
	6–24h		1.238	1.02–1.502	0.031
90‐day poor prognosis					
	≤ogno	1.154	1.051–1.268	0.003	
	6‐24h		1.503	1.18–1.916	0.001

### 3.5 Correlation Analysis of SHR With Adverse Outcomes in AIS Patients After Successful Recanalization by MT Across Different Blood Glucose Status

The study included 156 diabetic patients and 238 non‐diabetic patients, and there were 120 patients with HbA1c > 6.5% and 274 patients with HbA1c ≤ 6.5%. Patients were further stratified into subgroups based on adverse outcomes and prognosis. Rates of 3‐month outcomes in different blood glucose status groups (diabetes, non‐diabetes, HbA1c ≤ 6.5%, and HbA1c > 6.5%) are reported in Figure 2C,D.

The analysis showed that in the diabetes group, SHR was not significantly associated with HT or 90‐day death (*p* > 0.05). However, SHR was an independent risk factor for END, sICH, and poor prognosis after recanalization (*p* < 0.05). In the non‐diabetes group, SHR was an independent risk factor for END, HT, sICH, 90‐day death, and poor prognosis after recanalization (*p* < 0.05). Subgroup analysis indicated that in both the HbA1c > 6.5% and HbA1c ≤ 6.5% groups, SHR was an independent risk factor for END, HT, sICH, 90‐day death, and poor prognosis after successful recanalization (*p* < 0.05). Refer to Table 7 for detailed results.

**TABLE 7:** Multivariable regression analysis of SHR and adverse outcomes across different blood glucose status.

Outcome	Group	OR	95%CI	*p‐*value
HT				
	All patients	1.095	1.023–1.173	0.009
	Diabetes	1.032	0.932–1.143	0.542
	Non‐diabetes	1.169	1.053–1.297	0.003
	HbA1c ≤ 6.5%	1.097	1.016–1.185	0.018
	HbA1c > 6.5%	1.217	1.034–1.431	0.018
sICH				
	All patients	1.165	1.08–1.257	< 0.001
	Diabetes	1.182	1.039–1.345	0.011
	Non‐diabetes	1.153	1.043–1.275	0.005
	HbA1c ≤ 6.5%	1.126	1.038–1.222	0.004
	HbA1c > 6.5%	1.391	1.12–1.728	0.003
END				
	All patients	1.184	1.097–1.278	< 0.001
	Diabetes	1.251	1.086–1.441	0.002
	Non‐diabetes	1.133	1.030–1.247	0.01
	HbA1c ≤ 6.5%	1.131	1.043–1.227	0.003
	HbA1c > 6.5%	1.585	1.270–1.978	< 0.001
90‐day death				
	All patients	1.144	1.055–1.241	0.001
	Diabetes	1.129	0.986–1.294	0.079
	Non‐diabetes	1.125	1.007–1.255	0.037
	HbA1c ≤ 6.5%	1.148	1.042–1.265	0.005
	HbA1c > 6.5%	1.322	1.056–1.656	0.015
90‐day poor prognosis				
	All patients	1.220	1.116–1.336	< 0.001
	Diabetes	1.314	1.143–1.510	< 0.001
	Non‐diabetes	1.15	1.030–1.284	0.013
	HbA1c ≤6.5%	1.101	1.012–1.198	0.025
	HbA1c > 6.5%	1.784	1.391–2.288	< 0.001

## 4 Discussion

SHG encompasses both elevated blood glucose levels in non‐diabetic patients upon admission and worsening hyperglycemia in diabetic patients. SHG has been extensively studied in critically ill patients, including those with acute myocardial infarction, sepsis, severe trauma, and stroke (Vedantam et al. 2022). Animal studies have shown that ischemic injury in rats disrupts the glycolytic pathway, resulting in elevated ketone bodies, increased utilization of plasma triglycerides, reduced levels of glycolytic intermediates (lactate, pyruvate, and acetate), and elevated blood glucose (Baranovicova et al. 2018). Unlike diabetes‐related hyperglycemia, stress‐induced hyperglycemia arises following ischemic events and is linked to activation of the hypothalamic‐pituitary‐adrenal axis and the sympathetic nervous system in response to stress. Dysregulation of neuroendocrine hormones triggers a cascade of reactions, leading to elevated blood glucose levels. The main contributors to SHG include transient relative insulin deficiency and increased hepatic gluconeogenesis (Marik and Bellomo 2013).

Since SHG is a common biochemical alteration in critically ill patients, particularly those with stroke, and the intensity of the stress response increases with stroke severity, SHG may be associated with the severity of stroke (Duan et al. 2023; Merlino et al. 2020). In this study, the NIHSS score at admission was used to assess the severity of the condition. When comparing SHR levels in AIS patients across different severity levels, we found that patients in the very severe stroke group had significantly higher SHR. Multivariable ordinal logistic regression analysis indicated that SHR was an independent risk factor for stroke severity in AIS patients. Higher SHR was associated with greater stroke severity. Elevated SHG may reflect a more intense inflammatory response in critically ill patients, potentially contributing to stroke progression. Previous studies have shown that SHG is associated with an increased risk of stroke in patients with transient ischemic attack (Guo et al. 2023). Post‐ischemic SHG may serve as a predictor of death and poor functional recovery in AIS patients. A study by Liu Xin et al. (Liu et al. 2022) found that the SHR was associated with 3‐month neurological outcomes in patients with acute large vessel occlusion following MT. Patients with higher SHR had lower rates of successful recanalization and worse neurological outcomes. Our study further explored the relationship between SHR and short‐term prognosis in AIS patients after successful thrombectomy, demonstrating that patients with poor prognosis had significantly higher SHR levels than those with favorable prognosis. SHR was identified as an independent risk factor for poor 90‐day outcomes in acute AIS patients after successful recanalization. The potential mechanisms underlying these associations may include the following: (1) Direct damage to ischemic brain tissue: SHG may exacerbate brain injury by increasing anaerobic glucose metabolism, which generates large amounts of lactate. This lactate can accumulate in the brain, leading to intracellular acidosis and enhanced oxidative stress, further worsening cerebral ischemia. This disruption of neuronal metabolism in the ischemic penumbra can expand the infarct core and lead to irreversible damage (Maida et al. 2022, Rosso et al. 2015). (2) Promotion of thrombosis: SHG may induce abnormal platelet aggregation and a hypercoagulable state, increasing the risk of thrombosis (Huang et al. 2017; Chen et al. 2017; Gresele et al. 2003; Kito et al. 2018). (3) Blood‐brain barrier disruption: Increased oxidative stress and inflammation due to SHG can compromise the integrity of the blood‐brain barrier, aggravate cerebral edema, and worsen ischemic brain injury (Wada et al. 2018; Gray et al. 2013; Bémeur et al. 2005). (4) Impaired collateral perfusion: Animal studies have shown that post‐stroke hyperglycemia can impair cortical collateral blood flow, exacerbating brain damage in stroke models (Biose et al. 2020). These mechanisms may explain why elevated SHG can intensify brain injury in stroke patients. However, further basic research is needed to elucidate the molecular pathways linking SHG and stroke‐related brain damage.

In 2015, five landmark trials (MR CLEAN, ESCAPE, REVASCAT, SWIFT PRIME, and EXTEND IA) demonstrated the benefits of MT performed within a strict time window of ≤ 6 h from stroke onset (Tuo et al. 2022). The DEFUSE 3 and DAWN trials extended this window to 16 and 24 h, respectively, using advanced multimodal imaging (Wang et al. 2022; Rivera‐Aponte et al. 2015), significantly broadening the indications for thrombectomy. In this study, patients were stratified by time to intervention: an onset‐to‐puncture time of ≤ 6 h and a time of 6–24 h. Our analysis showed that SHR was not significantly associated with HT in either group. However, in the OTP time ≤ 6 h group, SHR was an independent risk factor for END. In contrast, there was no significant association between SHR and END in the OTP time 6–24 h group. This discrepancy may be attributed to the longer time from onset, potentially masking the detrimental effects of SHG on neurological function.

Most current research suggests that SHG is linked to worse clinical outcomes and poor prognosis in non‐diabetic AIS patients (Yao et al. 2023). However, the relationship between SHG and clinical outcomes in diabetic AIS patients remains contentious. Some studies report a significant association between high SHR and poor prognosis only in non‐diabetic patients, while in diabetic patients, high SHR does not correlate significantly with adverse outcomes (Wang et al. 2022). Merlino et al. (2022) found no significant association between SHG and clinical outcome measures (e.g., 90‐day poor prognosis, 90‐day death, symptomatic intracranial hemorrhage) in diabetic AIS patients who received intravenous thrombolysis. Li et al. (2020) noted no difference in the association between SHG and 90‐day poor prognosis in diabetic versus non‐diabetic AIS patients, although a significant correlation with 1‐year mortality was found only in non‐diabetic patients. Conversely, other studies have shown that high SHR is associated with increased in‐hospital mortality in diabetic AIS patients (Mi et al. 2022). In our study, SHR was significantly associated with sICH, END, and 90‐day poor prognosis across all patients, regardless of diabetes history. However, SHR was correlated with HT and 90‐day death only in non‐diabetic patients, with no significant association observed in diabetic patients. The differential outcomes under varying glycemic backgrounds may be attributed to chronic hyperglycemia‐induced pathological changes and increased tolerance to elevated glucose metabolism (Ma et al. 2020). Additionally, considering the potential presence of undiagnosed diabetes and well‐controlled HbA1c levels in some diabetic patients, we conducted a subgroup analysis based on HbA1c levels. The results indicated that SHR was significantly associated with adverse outcomes and 90‐day poor prognosis across all subgroups, irrespective of HbA1c levels. The differential outcomes under varying glycemic backgrounds may be attributed to chronic hyperglycemia‐induced pathological changes and increased tolerance to elevated glucose metabolism (Ma et al. 2020). Although patients with DM may exhibit a degree of “tolerance” to hyperglycemia, acute glucose elevation can be superimposed on chronic injury, leading to adverse outcomes (Capes et al. 2001). In nondiabetic patients, stroke‐induced stress triggers acute oxidative stress, which leads to microvascular rupture. The absence of chronic adaptation in nondiabetic individuals results in greater vascular fragility and heightened susceptibility to hemorrhage following reperfusion. In diabetic patients, the prolonged exposure to a chronic hyperglycemic environment induces structural remodeling of the vasculature (e.g., fibrosis and thickening of the basement membrane), potentially mitigating the additional disruption of vascular integrity associated with acute hyperglycemia (Horton and Barrett 2021; Yao et al. 2023). Diabetic patients typically exhibit poor collateral circulation. In such patients, who already have limited restoration of blood flow after reperfusion therapy and frequently develop comorbidities as a result of chronic hyperglycemia, the risk of HT and death is more likely driven by ischemic injury itself or by these comorbidities rather than by SHR. Furthermore, the majority of diabetic patients receive glucose‐lowering therapy, which may obscure the impact of SHR due to the presence of other complications. Further studies are warranted to clarify the mechanisms by which SHG influences clinical outcomes under different glycemic conditions.

This study has certain limitations. First, the data were collected from a single center, and the sample size was relatively small, with only 90‐day outcomes assessed. Larger, multicenter studies are needed to confirm our findings and evaluate long‐term prognosis. Second, patients without available FBG and HbA1c data were excluded, which may introduce selection bias. Future studies with expanded sample sizes are necessary to address these limitations.

## Author Contributions

**Xiaobo Li**: conceptualization, formal analysis, data curation, writing–original draft, and writing–review and editing. **Danni Chen**: methodology. **Chao Jiang**: validation. **Xianmin Wang**: formal analysis, data curation, writing–original draft, and writing–review and editing. **Wenyong Gao**: investigation and resources.

## Funding

This study was supported in part by grants from the National Natural Science Foundation of China (81371377), Jiangsu Provincial Department of Human Resources and Social Security Project Funding (2016‐WSN‐274), Jiangsu Province 333 Project Scientific Research Project Funding (BRA2017168).

## Conflicts of Interest

The authors declare no conflict of interest

## Ethics Statement

The studies involving human participants were reviewed and approved by Medical Ethics Committee of Northern Jiangsu People's Hospital (**Ethics Approval Number: 2024ky312**).

## Data Availability

The raw data supporting the conclusions of this article will be made available by the authors, without undue reservation.
